# Exploring the experiences and expectations of pharmacist interns in large general hospitals in China: from the perspective of interns

**DOI:** 10.1186/s12909-022-03591-5

**Published:** 2022-07-07

**Authors:** Xiali Yao, Xuedong Jia, Xiangfen Shi, Gang Liu, Yuwei Li, Xiaojian Zhang, Shuzhang Du, Jun Li, Zhao Yin

**Affiliations:** 1 Department of pharmacy, The First Affiliated Hospital of Zhengzhou University, Zhengzhou, Henan China; 2 Department of Pharmacology, School of Basic Medical Sciences, Guizhou Medical University, Guiyang, Guizhou China; 3 College of Pharmacy, Jiangsu Food and Pharmaceutical Science College, Jiangsu Huaian, China

**Keywords:** Hospital-based pharmacy internship (HBPI), Experience, Expectation, Qualitative study

## Abstract

**Background:**

Hospital-based pharmacy internship (HBPI) is critical for the transition from “pharmacy students” to “professional pharmacists”. This study explores the pharmacist interns’ experiences and expectations for HBPI from their personal experiences intending to provide references for future hospital pharmacy education reform and policy development.

**Methods:**

This is a multicenter qualitative study applying focus group discussions. Pharmacist interns were invited as participants from large teaching hospitals in Henan, China. A thematic analysis was conducted to qualitatively analyze this data. Nvivo 12 was utilized for data management and processing.

**Results:**

Three focus group discussions were conducted, involving 16 interns as participants. Three themes were summarized regarding interns’ expectations and experiences: (1) positive experiences of the HBPI; (2) negative experiences of the HBPI; (3) expectations and suggestions for the HBPI.

**Conclusion:**

This study finds that the HBPI improves the professional knowledge, professional skills, and core competencies of interns. Therefore, the HBPI is an important preparation and transition stage for pharmacy students. However, the current pharmacy internship in China still has imperfections such as the insufficient ability of clinical teachers, unreasonable internship models, and unscientific internship content.

**Supplementary Information:**

The online version contains supplementary material available at 10.1186/s12909-022-03591-5.

## Introduction

The increasing number of hospital pharmacists, coupled with a growing and aging population [1], provides a timely opportunity for hospital pharmacists in mainland China to become direct pharmaceutical care providers [2]. However, the current school-based pharmacy education in China is biased toward basic knowledge such as medicinal chemistry and pharmacology, and there is a great lack of training in clinical practice skills, making the hospital pharmacists unable to meet the demand for pharmacy services with their existing professional knowledge, skills, or competency [3]. Similar situations have happened in other Asian countries: although many hospital pharmacists have obtained a bachelor’s degree of science or doctor’s degree of pharmacy, they are considered unprepared to practice in most pharmacy-related settings [4, 5]. Thus, HBPI turns out to be a crucial period of adaptation and preparation for pharmacy students [4]. During the internship, pharmacy students as participants gain a concrete understanding of the hospital pharmacy profession and at the same time enhance their professional knowledge and skills. Hence, HBPI plays a key role in the transition from “pharmacy students” to “hospital pharmacists” [6]. Therefore, HBPI is particularly important for pharmacy students to bridge the gap between the preparation of knowledge from schooling and the demand for hospital pharmacy services.

Compared with the hospital-based pharmacy education in the USA, which has been developing for more than 60 years, the HBPI in China started late with a short history of about 30 years [3]. Early internships were mainly in the form of training courses, which focused on learning basic knowledge of hospital pharmacy and basic clinical practice skills [7]. Since the new century, the training content begin to involve clinical medical knowledge, such as anatomy and physiology, immunology, as well as clinical pharmacy practice skills [8]. In recent years, the HBPI in China has continued to learn from developed countries and gradually formed relatively mature programs in some large teaching hospitals [9]. As reported by a study conducted in Shanghai, the content of HBPI training has continued to vary to include the training of core competencies such as independent learning ability, career development ability, and leadership ability [10]. Clinical practical skills such as rapid identification of drug quality, use of automated equipment, pharmacy monitoring, patient consultation, therapeutic drug monitoring, and pharmacy knowledge including pharmacotherapeutics and clinical pharmacology were also given full consideration [10]. However, there are still some flaws with the current HBPI in mainland China: (1) there is a lack of nation-level operational guidelines; (2) there is no bidirectional evaluation tool for evaluating the effectiveness of internships; (3) the training content of the HBPI is not well integrated with the existing education activities, which could increase the burden of interns and lead to the accumulation of negative emotions. The above factors tend to affect the experiences and gains of pharmacist interns, which have long been significant reasons why the current HBPI can hardly meet the demand for pharmacy services [11–14].

Previous studies have shown that interns encounter significant difficulties during hospital internships. A recent study revealed that interns had a higher level of professional stress and more weekend work time, resulting in a higher prevalence of burnout [15]. A systematic review has also shown that clinical medical interns’ negative mindsets and experiences have a significant impact on the internship process [16]. Hence, the primary goal of the present study lies in exploring the interns’ feelings, especially their psychological experiences during the internship process. Meanwhile, positive experiences are important [17], which have voiced paradigms to approach burnout during hospital internship [16]. Positive psychology interventions such as gratefulness, savoring, forgiveness, and meaningful activities can achieve positive health and wellbeing [18]. Besides, teachers have been proved to play important roles in the teaching process of HBPI. A recent study showed that teachers helped interns at all stages of their learning, thereby significantly enhancing their core competencies such as independent learning ability; while irresponsible and indifferent clinical teachers would directly affect internship outcomes [19]. In short, interns’ negative or positive experiences and the intervention of teachers are key factors affecting the effectiveness of HBPI. However, to the best of our knowledge, there is no relevant research to understand the experience of Chinese pharmacist interns participating in HBPI. Their positive or negative feelings, their evaluation of the teachers of HBPI, or their suggestions for the development of the HBPI project have long been ignored.

Against this background, it’s necessary to understand how interns feel about HBPI from their own experiences. Therefore, a qualitative study was conducted, and Chinese hospital pharmacy interns were involved as participants. By exploring their feelings, expectations, and suggestions about HBPI, the present study aims to provide references for future hospital pharmacy education reform in China or other countries.

## Methods

### Ethical considerations

Ethics was approved by The First Affiliated Hospital of Zhengzhou University Institutional Review Board approved the protocol (2019-KY-304). Written informed consent was obtained from all the study participants.

### Sample and recruitment

Several large teaching hospitals in Henan province were selected. The sample hospitals were the First Affiliated Hospital of Zhengzhou University (approximately 8500 beds and 400 pharmacists), Henan Provincial People’s Hospital (approximately 5000 beds and 300 pharmacists), and the Third Affiliated Hospital of Zhengzhou University (approximately 2000 beds and 50 pharmacists). These hospitals are traditional teaching hospitals for medical students including pharmacy students, ranking top in central China. The hardware conditions available for students include drug dispensing rooms, pharmacy intravenous admixture centers, clinical pharmacology laboratories, and blood concentration monitoring centers, providing pharmacy interns with diverse internship options.

A purposive sampling method was used to select pharmacy interns as respondents from sample hospitals [20]. The inclusion criteria for respondents were as follows:

- working as an intern in one of the sample hospitals for at least 3 months
- finished three years of studying in the college of pharmacy at least
- willing to participate in the present study

The study was conducted near the end of the internship so that the interns would have a full understanding of the internship programs.

### Data collection

The interviews were conducted between February 2021 to June 2021. Invitations were sent to pharmacist interns who entered the pharmacy departments of sample hospitals in the fall of 2020. According to the respondents’ willingness, focus group discussions were organized [21]. Before the formal interviews, we communicated with the interns about the purpose of the study, the interview format, and the time and place for group discussions. No other people disturbed the interviews, and no clinical teachers were involved in the group discussions. The discussions were organized by one researcher (ZY) and the non-verbal interactions were recorded by another researcher (XLY). The discussion processes were audio-recorded.

In the first focus group discussion, the interview outline included interns’ gains, difficulties, and suggestions. During the first discussion, it was found that interns expressed significant negative experiences during the internship. Therefore, in the follow-up focus group discussions, attention to the interns’ psychological experiences were paid [20]. Data saturation was reached after three focus group discussions. Eventually, 16 interns participated in this qualitative study [21].

### Data analysis

Thematic analysis was used for the analysis of the interview data [18, 22]. Two researchers verbatim transcribed the audio recordings within 24 h of the group discussion. Then, two researchers (ZY and XDJ) independently extracted codes from the interview data and wrote the first draft of the themes and sub-themes. During this process, NVivo12 was used to manage and analyze the data. Subsequently, the research team discussed the framework of themes and language expressions. The final themes and subthemes were developed with the agreement of the research team, and appropriate representative quotes were selected to present themes or sub-themes. The report was conducted under the guidance of the Consolidated Criteria for Reporting Qualitative Studies (COREQ) checklist [23] (shown as supplementary material).

### Trustworthiness

The following measures were taken to ensure the trustworthiness of this study: (1) the research team consisted of hospital pharmacists or social scientists with extensive experience in conducting qualitative research, and close contact was maintained with the experts to continuously resolve difficulties during the study; (2) after processing the transcribed interview data, the researchers confirmed with the participants that the results of the study were consistent with what they wanted to convey; (3) the study was conducted in strict compliance with the operational procedures and methods.

## Results

**Fig. 1:**
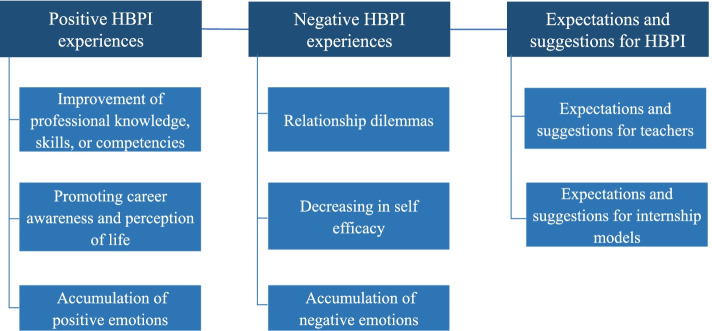
Three domains of the pharmacist intern’s experiences and expectations for HBPI

A total of 16 respondents were included in this study, their demographic characteristics are shown as Table 1. Three themes were identified: positive HBPI experiences, negative HBPI experiences, and suggestions for HBPI, the framework of themes and sub-themes is shown as Fig. 1.

**Table 1:** Overview of demographic characteristics

Demographic characteristics	Value
*Number of participants, n*	
group 1	6
group 2	5
group 3	5
*Sex, n*	
Female	15
Male	1
*Age, years*	
Mean (range)	21 (20–24)
Median	21
*Level of education, n*	Undergraduate or bachelor’s degree, 16
*Major*	Pharmacy
*Number of departments rotated, n*	
1 department	2
2 departments	5
3 departments	3
4 departments	1
5 departments	5
*Intership duration, n*	
6 months	4
7 months	2
8 months	10

### Positive HBPI experiences

The first theme was about interns’ positive internship experiences, including the following three sub-themes: (1) improvement of professional knowledge, skills, or competencies; (2) promoting career awareness and perception of life; (3) accumulation of positive emotions.

Most of the interns expressed that they got a chance to put knowledge into practice. Therefore, their professional knowledge got improved.

*1D: For example, the specific concepts of drugs. I learned their specific ingredients from the book, such as Atorvastatin calcium tablet. Now I know what it looks like. Before that, it was just a vague concept.*

Participants in our study said that their experimental skills and clinical pharmacy service skills were fully trained and improved through first-line laboratory or clinical practice.

2B: While in the lab, I learned about cell culture passaging and recovery, and the method of preparing cell culture fluids. Then, I also learned some other tricks about cell experiments.

*1D: Sometimes, I helped the teacher with his daily work. At that time, I had the opportunity to participate in the patient’s prescription review, medication consultation, case discussion, adverse reaction handling and reporting, which was very helpful to me.*

Participants stated that they improved their core competencies such as interpersonal communication or self-directed learning during the internship.

*1D:The first thing I feel is that I have improved my communication skills, especially when I go to ward for rounds. There are many things to pay attention to when communicating with patients, and this is a great opportunity to exercise your communication skills.*

*3B: I think, err…, I have developed the ability to learn on my own.*

Participants also mentioned the fostering of career awareness and perceptions of life.

*3D: Before (the internship), I was not so interested in my major (pharmacy). But now I have a clear goal. I am sure that I am interested in drug synthesis.*

*2B:It is mainly about the work attitude and the mentality of being an experimenter. I have learned to be careful and patient. Being a human being, um, just like doing experiments, you can’t be particularly timid. You have to do it boldly, and carefully. Yep.*

The participants stated that their professional knowledge and skills were improved, career awareness was fostered, resulting in an accumulation of positive emotions.

*1F: I felt that I was also useful, and my efforts were being recognized. The teachers also said that I did a good job this morning because I didn’t make mistakes when taking the medicine. I feel so joyful.*

*2E: Later on, I became faster, and I was getting to know more. After that, I found that it was not so difficult. Actually, it was quite simple.*

### Negative HBPI experiences

Regarding negative HBPI experiences, respondents highlighted three aspects: relationship dilemmas, decreasing in self efficacy, and accumulation of negative emotions. Many respondents expressed great concerns about the interpersonal relationships: teacher-student relationships and student–student relationships, resulting in significant relationship dilemmas.

*1F: But the three of us were worried, just because our teachers had their own projects. They were always busy with their own work. We were afraid to disturb or bother them, and we were also very embarrassed to talk to them.*

*1C: I don’t communicate enough with my teacher. Maybe it’s because my teacher is shy, and I am also shy. Then we just don’t communicate so much like others do.*

*2A: Because of the changes in the environment, my relationship with some of my classmates became very tense.*

Some participants felt a clear decrease in self efficacy. It was stated that the lack of professional knowledge affected their confidence, and they were unable to keep up with their teachers’ teaching pace during the internship.

*1E: Because of the lack of knowledge, my first reaction to new things is fear. I am afraid that I can’t do it well or I don’t know it at all.*

*1D: Sometimes I feel that I can’t follow the teachers. I want to help, but I was not able to do so.*

Interns reported difficulties related to the clinical teachers, such as negative attitude or lack of communication, which led to the accumulation of negative emotions and made the interns suffer from powerlessness, sadness, and exhaustion.

*1B: Some teachers give me the feeling that I am a medicine-taking machine in the pharmacy room. They never communicate with us.*

*1F: Because I felt that all kinds of pressure were pressed together, it was very uncomfortable at that moment.*

*1E: At noon that day when she got off work, she felt aggrieved and cried while talking.*

### Expectations and suggestions for HBPI

Based on the experiences of the internship process, the interns expressed their expectations and suggestions for teachers and teaching models. Regarding teachers, interns reported that the teachers should apply more efficient ways to manage the internship process. In addition, the interns expected their teachers to guide and career planning according to interns’ personal characteristics and interests. Meanwhile, communications in a convenient and comfortable way between interns and teachers were highlighted by nearly all the participants.

*1B: As fresh graduates, we all have the same feelings that we need someone to guide us. There are so many problems that we can’t solve by ourselves.*

*2B: The teacher needs to be able to communicate with students: listening to their own ideas, their future goals, and giving advice.*

*3C: They might give us some guidance on life, and we would have more career options to choose that fit us when we graduate.*

*1F: It just lacks a leader or model. Because teachers are more experienced than us, or they may encounter similar problems to us. For me, teachers are often role models.*

Meanwhile, the participants stated their suggestions on teaching models mainly included a full preparation for entry into a new department, reasonable rotation plans, and standardization of training contents.

*1B: Then I think. Er, I very much hoped that before entering a new department, I had a more comprehensive understanding of it, so that I could better carry out the internship.*

*3B: I thought what needs to be improved was a more reasonable rotation plan, which could comprehensively improve my knowledge and ability.*

*2A: For me, I thought the standardization of internship content was an urgent matter.*

## Discussion

To our knowledge, this is the first qualitative study in China to explore the experiences of pharmacy interns in hospitals. As a preliminary analysis of the reasons for the accumulation of their positive or negative emotions, the findings of the present study provide suggestions and references for future improvements in HBPI programs. It’s revealed that the current HBPI is important for pharmacy students to adapt and prepare for their careers in advance. However, there are obvious flaws in internship programs in China.

Previous studies have shown that HBPI improved pharmacy students’ professional knowledge, skills, career awareness, and personal perception of life [10]. The results of this study also showed that HBPI gave pharmacist interns the opportunity to participate in prescription review, medication consultation, case discussion, adverse reaction handling and reporting. The process of linking theory with practice helped them improve their professional knowledge and skills [24]. Along with the improvement of their knowledge and skills, they also had a clearer understanding of the profession, and most of the respondents said that they gained a sense of accomplishment during the internship, which enhanced their love for their career [25, 26]. Meanwhile, receiving positive affirmations from their teachers stimulated their interest in learning. Besides, communication with teachers or patients, their communication skills were significantly improved [27]. However, it is worth pointing out that the traditional core competency evaluation index system for pharmacists [28] requires that hospital pharmacists should be health care providers combining several competencies. Unfortunately, the benefits of the internship mentioned by the interns in this study did not include the enhancement of leadership skills, management skills, decision-making skills, and critical thinking, which are areas that need to be improved for future pharmacy internships.

A meta-analysis conducted by Erschens, R et al. shows that 7.0% to 75.2% of pharmacy students have experienced work-related burnout [29], and the reason might be a lack of clinical knowledge and skills, as well as poor relationships with colleagues or heavy workload [30]. In the present study, respondents also mentioned many negative experiences, including relationship dilemmas, decreasing in self efficacy, and accumulation of negative emotions. A study highlighted interpersonal relationship dilemmas during the internship process, and this study also pointed out that the interns were concerned about interpersonal relationships such as colleague relationships and student–teacher relationships, resulting in inadequate communication [31]. In addition, the interns with insufficient professional knowledge were not able to keep up with their teachers’ teaching pace during the internship, resulting in an obvious sense of powerlessness [32]. Moreover, the participants pointed out that the lack of interaction with students might cause negative feelings among them. Similarly, the monotonous internship content and teaching models led to exhaustion and burnout among interns, which is worth further consideration [33, 34].

In response to the difficulties faced and negative emotions experienced during the internship, as well as the knowledge and positive experiences gained, the interns provided suggestions and expectations for the teachers and teaching models. Pharmacy education is characterized by the fact that much of the educational process takes place in the course of clinical practice, where clinical teachers play pivotal roles [35]. The internships are influenced in a positive or negative way by the behavior of clinical teachers during daily practice [36, 37]. The results of this study showed that clinical teachers were role models and interns could enhance learning effectiveness by observational learning, which fits the framework of the Social Learning Theory [38]. Irby D [39] described in detail the role modelling of clinical teachers: “they are faculty members who demonstrate clinical skills and articulate expert thought processes and manifest positive professional characteristics”, which highlighted the roles of teachers in building internship plans and promoting two-way communication. Previous research explored the construction of a rational teaching model to support clinical learning and collegial relationships building [40]. It is well known that new types of teaching approaches such as Problem-based Learning (PBL) or Case-based Learning (CBL) help to improve the effectiveness of the internship [41]. In terms of theoretical knowledge learning, PBL helps students enhance their interest in learning and improve their self-learning abilities [42]. CBL helps to improve students’ analytical and decision-making skills [43]. The process of case discussion will also significantly improve students’ cooperation, communication, and expression skills. Combined with the results of this study, these new types of teaching methods should be applied in HBPI in China and other countries to meet students’ expectations. Since interest has always been an important motivation for learning [44], the internship models should fit the interests of interns.

There are several limitations in this study. Firstly, given the important roles of clinical teachers, this study did not involve teachers as participants. It’s necessary to conduct research on the experiences of clinical teachers to analyze HBPI in a more comprehensive way in the future, which has been planned as the next research plan of our research group. In addition, the research participants are all from central China and the study has limited enrollment. The next research plan consists of the following: (1) using the Delphi method to explore the training needs for pharmacy students from HBPI or to construct the syllabus of HBPI’s content; (2) applying qualitative study to explore expectations and suggestions from the perspective of teachers; (3) conducting multi-center studies include more pharmacy interns and clinical teachers to further verify the findings of the present study.

## Conclusion

HBPI is an important preparation and enhancement stage for pharmacy students in the transition to full-time hospital pharmacists, and this process can significantly enhance the professional knowledge, professional skills, and core competencies of interns. However, there exist some flaws in HBPI in mainland China such as inadequate competence of clinical teachers, unreasonable internship models, and unscientific internship content. Future HBPI programs should pay attention to the following issues: (1) taking the core competency model of pharmacists as a reference to design internship content; (2) in terms of teaching methods, introducing diversified methods such as PBL or CBL; (3) taking systematic measures to improve the teaching abilities of clinical teachers; (4) and constructing scientific evaluation mechanisms and tools to bidirectionally evaluate the effectiveness of internships.

## Supplementary Information

**Additional file 1.** The checklist of the research. 

**Additional file 2:** **Box 1.** Questions used in the interview guide.

## Data Availability

The datasets used and/or analyzed during the current study are available from the corresponding author on reasonable request.
