# Supplementary material for: Exploring the experiences and expectations of pharmacist interns in large general hospitals in China: from the perspective of interns
Source: BMC Med Educ. 2022 Jul 7;22:528. doi: 10.1186/s12909-022-03591-5 (PMC9264576; doi:10.1186/s12909-022-03591-5)
Supplement: Supplementary file 2 — Additional file 2: Box 1. Questions used in the interview guide. [file 12909_2022_3591_MOESM2_ESM.docx]

**Box 1. Questions used in the interview guide**

We want to know your feelings about the internship. Your experiences and suggestions are of great significance for improving the pharmacy internship program in the future. Thanks for your participation!

**Q1 The first question I want to talk about is, what are the positive memories you have during the whole internship?**

- What do you think is your greatest achievement?
- Please give an example to talk about your gains, please?
- What do you think is the experience of going to the ward for rounds or prescription review with the. teacher?

**Q2 Please recall what negative memories you have during the whole internship?**

- Please recall what regrets do you feel during the internship?
- There are difficulties, right? Please tell us something in detail.
- Do you also experience burnout? What factors cause burnout?

**Q3 Based on your personal experience, do you have any suggestions for the pharmacy internship program?**

- Suppose you were asked to design a pharmacy internship program, how would you design it?
- In order to better improve the effect of the internship, what would you like to say to your teachers?
- What other suggestions do you have for improving this internship?
